# Euglycemic Diabetic Ketoacidosis in Type 1 Diabetes on Insulin Pump, with Acute Appendicitis: A Case Report

**DOI:** 10.5811/cpcem.2021.1.48905

**Published:** 2021-06-29

**Authors:** Brian D. Thompson, Anthony Kitchen

**Affiliations:** University of Massachusetts Medical School - Baystate Health, Department of Emergency Medicine, Springfield, Massachusetts

**Keywords:** Euglycemic diabetic ketoacidosis, emergency medicine

## Abstract

**Introduction:**

Recently, euglycemic diabetic ketoacidosis has been an increasing topic of discussion within emergency medicine literature. Euglycemic diabetic ketoacidosis can easily be missed, as a normal point-of-care glucose often mistakenly precludes the work-up of diabetic ketoacidosis.

**Case Report:**

A 16-year-old female with a past medical history of type 1 diabetes presented to the emergency department with altered mental status, vomiting, and abdominal pain. She was diagnosed with euglycemic diabetic ketoacidosis.

**Conclusion:**

Reported cases of euglycemic diabetic ketoacidosis are most frequently attributed to sodium glucose cotransporter-2 inhibitors, but other potential causes have been discussed in the literature. In this patient, a starvation state with continued insulin use in the setting of acute appendicitis led to her condition.

## INTRODUCTION

Euglycemic diabetic ketoacidosis (eDKA) is a clinical syndrome that can occur in patients with type 1 or type 2 diabetes. It is characterized by euglycemia, defined as blood glucose less than 250 milligrams per deciliter (mg/dL), in the presence of severe metabolic acidosis, defined as arterial pH less than 7.3 and serum bicarbonate less than 18 milliequivalents per liter (mEq/L) with ketonemia.^1^ This condition was first described in 1973. Recent case reports suggest an increase in presentations due to increased use of sodium glucose cotransporter-2 (SGLT2) inhibitors.^2^–^11^ Sodium glucose cotransporter-2 inhibitors work by blocking glucose reabsorption of filtered glucose in the proximal tubule and subsequently lowering serum glucose.

The initial laboratory values of a patient presenting with eDKA may mislead clinicians, as over-reliance on point-of-care glucose testing, and the finding of a normal blood glucose, may cause clinicians to prematurely contract their list of differential diagnoses in an undifferentiated patient with altered mental status. Initial glucose of less than 250 mg/dL (reference range 80–140 mg/dL) may delay the diagnosis of eDKA or lead the clinician down a differential of elevated gap metabolic acidosis without considering DKA as the primary metabolic derangement. This may prompt unnecessary testing and treatments that could delay diagnosis and definitive treatment. Cases of eDKA reported in the literature are most often attributed to SGLT2 inhibitors, but occasional cases have been reported involving starvation states, pregnancy, cocaine abuse, prolonged vomiting, diarrhea, and insulin use.^3^ Emergency physicians should not only be aware of this diagnosis but also potential causes other than SGLT2 inhibitors, as delays in the diagnosis of DKA can cause substantial morbidity and mortality.

## CASE REPORT

The patient was a 16-year-old female with a past medical history of type 1 diabetes on insulin pump therapy, previous episodes of DKA, and autoimmune hypothyroidism. She presented to the emergency department with altered mental status, severe abdominal pain, and multiple episodes of vomiting. According to her mother, all her symptoms started within the prior 24 hours. The patient was observed to be awake but disoriented with generalized abdominal tenderness to palpation. The insulin pump was taken off by the family just prior to presenting to the hospital. She presented to the hospital via emergency medical services, and her vital signs were normal upon arrival. Initial physical examination revealed a Glasgow Coma Scale (GCS) of 12 (eyes 3, verbal 4, motor 5) and the patient was localizing to pain on palpation of the right lower quadrant, without any other significant exam findings. Monitoring and intravenous (IV) access were established, and blood for laboratory evaluation was collected.

The patient was started on a lactated Ringer’s infusion. Her vital signs remained stable and upon re-evaluation, her GCS remained unchanged. With her clinical picture and previous history with DKA, initial concern was for DKA, although her initial point-of-care glucose was 109 mg/dL (reference 80–140 mg/dL) Subsequent point-of-care glucose readings showed decreasing levels less than 100 mg/dL (80–140 mg/dl) requiring glucose administration. Because of initial normal point-of-care glucose readings, the treating team pursued other causes of altered mental status. Initial labs were significant for a venous pH of 7.2 (7.35–7.45), bicarbonate of 8 mEq/L (21–28 mEq/L); anion gap of 33 millimoles per liter (mmol/L) (4–12 mmol/L); lactate of 2 mmol/L (0.5–1 mmol/L); sodium 133 mEq/L (135–145 mEq/L); potassium 4.9 mEq/L (3.6–5.2 mEq/L); blood urea nitrogen 19 mg/dL (7–20 mg/dL); creatinine of 0.8 mg/dL (0.84–1.21 mg/dL); beta-hydroxybutyrate of 3.26 mmol/L (0.4–0.5 mmol/L); initial plasma glucose of 108 mg/dL (80–140/mg/dL); and leukocytosis of 20,600 per microliter (4500–11000 per microliter).

Urine toxicology, salicylate, and acetaminophen screen and pregnancy test were negative. A non-contrast computed tomography (CT) of the brain was unremarkable. She also had an IV contrast-enhanced CT of the abdomen, which was suspicious for possible early appendicitis. With her resulting labs, other causes of elevated gap metabolic acidosis were considered. An endocrine consult was obtained for possible eDKA. Based on the consult and test results, the patient was started on an insulin drip at 0.1units per kilogram per hour as well as an infusion of a dextrose 10% solution.

Pediatric surgery was consulted, and after exam and review of the CT results recommended that the patient be taken to the operating room for an appendectomy. Surgical findings were noted to be equivocal and the appendix was removed without complication. The patient’s mental status improved post appendectomy. With fluid and insulin therapy her condition improved, laboratory abnormalities normalized within a few days, and she was subsequently discharged home. The consulting teams agreed that the final diagnosis was eDKA likely caused by appendicitis.

## DISCUSSION

A literature review of this topic reveals mostly case reports. In the published reports, most patients with eDKA had been taking SGLT2 inhibitors, which were thought to have precipitated the eDKA. Very few case reports recognized other causes of eDKA. Approximately 2.6–3.2% of DKA admissions are euglycemic, although cases of eDKA may be under-reported.^1^,^14^,^15^ The exact mechanism of eDKA is not entirely known. It is proposed that insulin-dependent diabetics in a fasting state can develop severe ketoacidosis without pronounced hyperglycemia as a consequence of a carbohydrate deficit. This results in decreased serum insulin and excess counter-regulatory hormones such as glucagon, epinephrine, and cortisol. The increased glucagon/ insulin ratio leads to increased lipolysis, increased free fatty acids, and ketoacidosis. This state leads to decreased gluconeogenesis and enhanced cellular utilization of the limited available glucose.^2^

**CPC-EM Capsule**

What do we already know about this clinical entity?

*Euglycemic diabetic ketoacidosis (DKA) is a clinical syndrome first described in 1973. There has been an increase in presentations due to the increased use of sodium-glucose cotransporter-2 (SGLT2) inhibitors*.

What makes this presentation of disease reportable?

*Reported euglycemic DKA cases are most often attributed to SGLT2 inhibitors, but occasional cases have involved starvation states, as this one*.

What is the major learning point?

*Emergency physicians should be aware of this diagnosis and potential causes other than SGLT2 inhibitors, as delays can lead to morbidity and mortality*.

How might this improve emergency medicine practice?

*Clinicians should be able to recognize and treat Euglycemic DKA and be avoid the pitfall of using normal blood glucose to rule out DKA*.

The liver, in theory, will enter into a state of glycogen depletion and due to the fasting state, lipolysis and fatty acid production will also occur, causing excessive ketone body production resulting in acidemia with euglycemia.^2^,^12^,^13^ This is in contrast to starvation ketoacidosis, where the clinical course is usually more insidious and the bicarbonate level is usually greater than18 mEq/L (21–28 mEq/L). Presumably, in our patient’s case, starvation and vomiting in the setting of early appendicitis served as a triggering event for eDKA. Her vomiting, fasting state, and continued use of insulin contributed to glycogen storage depletion causing euglycemia and even mild hypoglycemia.

Although the patient was still using insulin, presumably the ratio between glucagon and insulin was altered because as her serum glucose remained low, her injected insulin remained at her basal rate. Her family then removed the insulin pump just prior to arrival, further decreasing her insulin levels. In this state lipolysis and fatty acid production were upregulated, which led to excessive ketone body production. And with her brief period of illness, she was still in a state of relative euvolemia. In terms of treatment, a patient may require more IV dextrose than hyperglycemic DKA patients in order to maintain a euglycemic state, since treatment in both DKA and eDKA requires administering fluids and continuous IV insulin.

## CONCLUSION

Diabetic ketoacidosis is commonly diagnosed and treated by emergency clinicians, but euglycemic diabetic ketoacidosis is less frequently encountered. Clinicians should be able to both recognize and treat eDKA, and be aware of the pitfalls of using a euglycemic blood glucose alone to rule out the possibility of DKA. In patients who potentially have diabetic ketoacidosis but have normal glucose levels, clinicians should consider continuing the work up for eDKA, particularly in patients with common potential risk factors such as treatment with SGLT2 inhibitors or a history of recent starvation or vomiting.
